# Magnitude and associated factors of diabetic complication among diabetic patients attending Gurage zone hospitals, South West Ethiopia

**DOI:** 10.1186/s13104-019-4808-9

**Published:** 2019-11-29

**Authors:** Bereket Beyene Gebre, Zebene Mekonnen Assefa

**Affiliations:** 1 School of Nursing, College of Health Science, Hawassa University, Hawassa, Ethiopia; 2 Department of Nursing, College of Health Science, Wolkite University, Wolkite, Ethiopia

**Keywords:** Diabetic complications, Magnitude, Associated factor

## Abstract

**Objective:**

To assess the magnitude of diabetic complication and associated factors among diabetes mellitus patients attending in Gurage zone hospitals.

**Results:**

According to this study the magnitude of diabetic complication among diabetic patients were 61% and the marital status; divorced [AOR: 0.252 (0.11, 0.59); p = 0.002], poor glycemic control [AOR: 1.88 (1.04, 3.39); p = 0.036], body mass index > 25 [AOR: 4.42 (1.32, 14.86); p = 0.016] and duration of illness > 6 years [AOR :1.79 (1.02, 3.17); p = 0.044] and 10 years [AOR: 4.68 (2.07, 10.61); p = < 0.001] were significantly associated with diabetic complication.

## Introduction

### Background

Diabetes is a chronic disease marked by high level of blood glucose that occurs either when the pancreas does not produce enough insulin or when the body cannot effectively use the insulin that was produced. It is one of the chronic non communicable diseases which have emerged as a leading global health problem [1]. In Africa it was estimated that 15.5 million adults aged 20–79 years were living with diabetes as according to International Diabetic Federation report (IDF) [2]. Out of the estimated cases 69.2% of adults were living with DM but they are unaware of their condition. Ethiopia is one of the 32 countries of the IDF African region report. There were 2,567,900 cases of diabetes in adults in Ethiopia in 2015 [3]. According to studies done in Jimma University specialized hospital report the number of people with diabetes has risen from 108 million in 1980 to 422 million in 2014 [4].

The seriousness of the disease is due to its complication. Complication of DM account for increased morbidity, disability and mortality and threats for the economies of all countries especially in the middle and low economic countries [5]. In 2016, an estimated 1.6 million deaths were directly caused by diabetes. Another 2.2 million deaths were attribute to high blood glucose in 2012 [1].

Based on the time required to develop complication diabetes has been classified in acute and chronic ones. Acute complication consists diabetic ketoacidosis which is the most common acute complication and it mostly occur in type 1 diabetes mellitus (DM). Hyperglycemic hyperosmolar non ketosis syndrome mostly occurs in type 2 DM and hypoglycemia [6, 7]. The chronic complications are broadly classified in to macro vascular, micro vascular and other complication of DM. Macro vascular complication of DM are primarily disease of coronary artery, cerebrovascular, and peripheral vascular. Microvascular complication of DM is those that affect small blood vessels typically include retinopathy, neuropathy and nephropathy. Other complication of DM is bacterial and fungal infection which occurs due to direct effects of hyperglycemia on cellular immunity [8]. DM can result in an increased risk of sever sight impairment, end stage kidney disease, cardiovascular disease and in some cases early death. In type 2 diabetes 75% of people will die of heart disease and 15% of stroke. The mortality rate from cardiovascular disease is up to five times higher in people with diabetes than in people without diabetes. The risk of death from diabetes related cause increased by 21% [9]. Since there is limited study in the study area, this study finding addresses to determine the magnitude of DM complication with its associated factor in the study area.

#### General objective

To assess magnitude and associated factors of diabetic complication among diabetic patients attending Gurage zone hospitals, Gurage, South West Ethiopia, from April 1 to May, 30, 2019.

#### Specific objective

- To determine the magnitude of diabetic complications among diabetic patients attending Gurage zone hospitals.
- To determine associated factors of diabetic complication among diabetic patients attending Gurage zone hospitals.

## Main text

### Methodology

#### Study area and period

This study was conducted at Gurage zone hospitals which is located in Southern Nations Nationalities and Peoples’ Region state (SNNPRs) of Ethiopia. The “Gurage” zone has three primary and one general hospital and 72 health centers. All hospitals have chronic follow up clinic especially for diabetes mellitus and hypertension. Those four hospitals were “Attat” (private hospital), “Gunchire”, and “Bui” are primary hospitals and Butajira is the only General hospital in the zone. The study was conducted from April 1 to April 30, 2019 G.C.

#### Study design

Institutional based cross sectional study was conducted.

#### Source population

All diabetic patients who were on follow up at Gurage zone hospitals.

#### Study population

All sampled diabetic patients attending “Butajira” general hospital, “Attat” and “Gunchire” primary hospitals follow up clinics during the study period.

#### Inclusion and exclusion criteria

##### Inclusion criteria

All diabetic patients who had follow up in Gurage zone sampled hospitals and were on follow up during the study period were included in the study.

##### Exclusion criteria

Patients who were newly diagnosed for diabetes mellitus and on treatments for less than 3 months durations were excluded.

Patients who were unconscious and had mental illness during data collections were excluded.

#### Sample size and sampling technique

Sample size was determined by using population formula.

$$n = \frac{{Z\left( {\frac{a}{2}} \right)^{2} P\left( {1 - P} \right)}}{{D^{2} }}$$

n—minimum sample, P—estimate % of diabetic complications among diabetic patients, D—margin of error (0.05), $Z(a/2)$—standard normal

$$n = \frac{{(1.96)^{2} \times 0.525\left( {1 - 0.525} \right)}}{{(0.05)^{2} }} = 384$$

Since the source of populations were 1540 which were below 10,000, the finite population correction is needed:

$$nf = \frac{n}{1 + n/N} = \frac{384}{1 + 384/1540} = 305$$

where N = total population.

The result after the correction became 307 and adding non response rate of 10% (307 × 10% = 30.7) of the total sample size; 30.7 + 307 = 338.

#### Sampling techniques

**Fig. 1:**
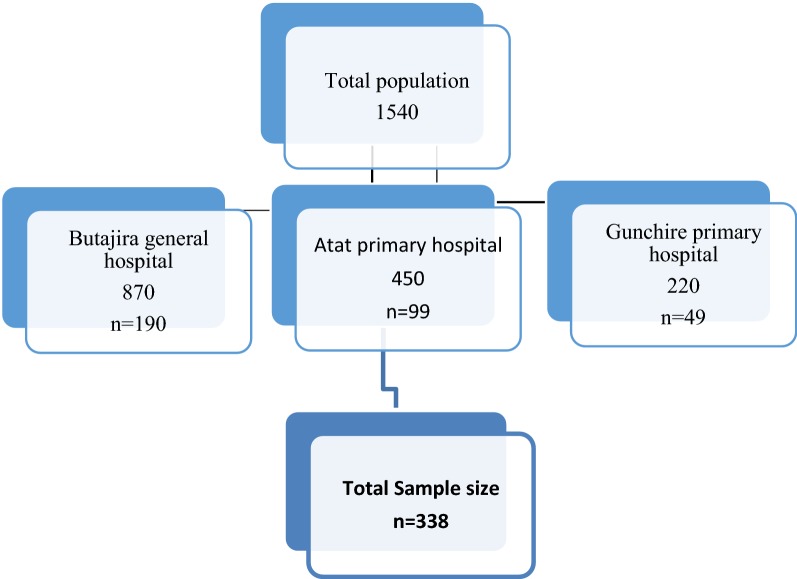
Schematic presentation of sampling procedure, Gurage zone hospitals in Ethiopia

Stratified sampling method was used to select the study sample from selected hospitals. First one general hospital was purposively selected and two primary hospitals were selected by random sampling method from three primary hospitals. Then stratified sampling and proportional allocation was done (see Fig. 1).

#### Study variables

##### Independent variables

- Socio-demographic characteristics (age, sex, weight, educational status, place of residence, income).
- Medical conditions [type of DM, hyperlipidemia, duration of disease, BMI, infection, other comorbidities (hypertension, heart diseases)].
- Type of medication [treatment adherence, insulin, oral hypoglycemic agent, and life style medication (diet, exercise), smoking].

##### Dependent variables

Diabetic complication.

#### Data collection procedure (instruments, personnel and data quality)

##### Data collection instruments

The data were collected using structured questionnaire and by reviewing patients cards for laboratory results and other medical findings. The questionnaire had three parts. These includes socio-demographic data, medical related factors and management parts.

##### Data collection process

Three diploma nurses were selected for data collection for each hospital with two B.Sc nurses for supervisors. Training were given for both data collectors and supervisors for 2 days before the actual data collections about the study procedure and data collection techniques.

#### Operational definition and definition of terms

##### Diabetic control

Good  ≤ 130 or RBS ≤ 200 and

Poor > 130 of FBS/RBS > 200.

##### Diabetic complication

No: if the diabetic patients have neither acute nor chronic complication.

Yes: if the patients has one complication either acute (hypoglycemia, DKA and HHNS) or chronic complication (Macro vascular, micro vascular, neuropathy, nephropathy or diabetic foot ulcer) that rule out by laboratory investigation and physical examination during follow up.

#### Data quality management

To assure the quality of data, properly designed data collection tool (structured questionnaire) was prepared in English and translated to Amharic language and then back to English to check the consistency by fluent English speaker (English language teacher) and finally the data were collected by English language. The questionnaire was pre tested on 5% [17] diabetic patients at in “Buiprimary” hospital follow up clinic to check the accuracy and validity of the questionnaire. Training was given for both data collectors and supervisors. Then data were checked and entered into EPI data version 3.1

#### Data processing and analysis

After data collection each questionnaires’ was checked visually for completeness. The responses were entered into EPI data Version 3.1 and exported to SPSS version 23 for clearing, coding and analysis. Both descriptive and analytic analysis was performed. Those explanatory variable with p-value < 0.2 at 95% CI in bivariate were eligible for multivariate and those variables with p value ≤ 0.05 were significant in multivariate at 95% CI.

#### Ethical consideration

Before data collection, Ethical clearance letter was obtained from Wolkite University College of medicine and health science department of nursing and dispatch to each selected hospitals and Gurage zonal health office. The respondents were informed and their written consent was obtained. The respondents had the right to refuse or with draw from participating in the interview at any time were respected and the information provided by each respondents were kept confidentially, each questionnaire were coded and information were not shared to the third party.

#### Dissemination of finding

The result of the study was disseminated to Wolkite University College of medicine and health science, department of nursing, Butajira general hospital, Attat primary hospital and Gunchire primary hospitals.

## Results

From a total of 338 diabetic patients included in the study, the response rate was 100%. Among the total respondents, 192 (56.8%) were male.

Among the total participants 155 (45.9%) used oral hypoglycemic agent, 160 (47.3%) used insulin only and 23 (6.8%) were used both oral hypoglycemic agent and insulin. Those who adhered to physical activity were 222 (65.7%) and 245 (72.5%) ate recommended dietary practice. From the participants 59.2% had taken their daily dose properly and 39 (11.5%) had smoke cigarette in addition to anti diabetic drugs. Besides, 160 (47.3%) were type 1 diabetes mellitus and 178 (52.7%) were type 2 diabetes mellitus, 150 (44.4%). Moreover; 27 (8%) were under weight, and 34 (10%) were overweight. Regarding their blood glucose level; 274 (81.1%) had RBS ≥ 200 mg/dl, 303 (89.6%) had FBS ≥ 130 mg/dl. From the participants; 168 (49.7%) had low density lipoprotein (LDL) ≥ 130 mg/dl, whereas, 82 (24.3%) had high level lipoprotein (HDL) ≥ 60 mg/dl.

Regarding acute complication of diabetes mellitus; 48 (14.2%) had DKA, 21 (6.2%) had HHNS and 22 (6.5%) had hypoglycemia. But from chronic complication 50 (14.8%) had neuropathy, 49 (14.5%) had visual impairment (retinopathy) and 57 (16.9%) had diabetic foot ulcer, 69 (20.4%). From the total of DM patients who had complication; 91 (26.9%) had acute complication whereas 205 (60.7%) had chronic complication (see Table 1).

**Table 1:** Independent predictors for diabetes complications among diabetes mellitus using forward binary logistic regression at Gurage zone hospitals, southwest, Ethiopia (n = 338)

Variables	DM complication		COR (95%) CI	AOR (95%) CI	p-value
	No	Yes			
Marital status					
Married	81 (60.9%)	150 (73.2%)	1:00	1:00	1:00
Single	33 (17.3%)	19 (9.3%)	0.45 (0.29, 0.87)	0.93 (0.43, 2.02)	0.931
Divorced	13 (9.8%)	21 (10.2%)	0.87 (0.42, 1.83)	0.523 (0.23, 1.18)	0.120
Widowed	16 (12.0%)	15 (7.3%)	0.51 (0.24, 1.17)	0.252 (0.11, 0.59)	0.002*
Glycemic control					
Good	102 (76.7%)	113 (55.1%)	1:00	1:00	1:00
Poor	31 (23.3%)	92 (44.9%)	2.68 (1.65, 4.36)	1.88 (1.04, 3.39)	0.036*
BMI					
< 18.5	17 (12.8%)	10 (4.9%)	1:00	1:00	1:00
18.5–24.9	108 (81.2%)	169 (82.4%)	2.66 (1.174, 6.03)	2.26 (0.91, 5.65)	0.08
≥ 25	8 (6.0%)	26 (12.7%)	5.53 (1.82, 16.8)	4.42 (1.32, 14.86)	0.016*
Duration of illness (years)					
< 5	79 (59.4%)	71 (34.6%)	1:000	1:00	1:00
6–10	42 (31.6%)	73 (35.6%)	1.93 (1.18, 3.18)	1.79 (1.02,3.17)	0.044*
> 10	12 (9%)	61 (29.8%)	5.65 (2.82, 11.34)	4.68 (2.07, 10.61)	< 0.001*

*Shows significant association

## Discussion

This study showed that the magnitude of DM complication among diabetic patients in Gurage zone hospitals was 61% and it was relatively similar with study done in Northeast Ethiopia (59.7%) and Bahirdar (53.5%) in Ethiopia [10, 11]. So, designing strategy for early diagnose and management of DM should be crucial.

In this study finding; DM patients who live with DM between 6 and 10 years and above 10 years were 1.79 and 4.68 times more likely to develop diabetes complication respectively when compared with patients who had live with DM less than 5 years duration. This study was similar with study conducted in Felegehiwot referral hospital, Bahirdar, Arbaminch and Jimma [10, 12, 13]. So, patients should be clinical managed properly by initiating early diagnose and treatment.

Regarding body mass index in our study; those diabetes mellitus patients who had higher BMI (>25 kg/cm^2^) had 4 times [AOR: 4.42, 95% CI (1.32, 14.86)] more likely to develop diabetic complication when compared with patients who had BMI < 18 kg/cm^2^. It was similar with study done in China [14] and Pacific Islands [15]. This will imply that having recommended dietary practice and exercising physical activity regularly will be strengthened.

In this study; those patients who were widowed marital status were 75% [AOR: 0.252; 95% CI (0.11, 0.59)] less likely to develop diabetic complication when compared with patients who had married marital status and it was similar with study done in Iran (0.74; 0.56–0.97) [16].

This study finding suggest that those patients who had poor glycemic control were 2 times [AOR: 1.88, 95% CI (1.04, 3.39)] more likely to develop diabetic complication when compared with patients who had good glycemic control. This study finding was similar with study done in Arbaminch and Gonder [12, 17]. So, continuous follow up and checking patients FBS should be taken thoroughly.

## Conclusions

The magnitude of diabetic complications among the Gurage zone hospitals was 61%. Being widowed, long duration of illness, having BMI greater than 25 kg/cm^2^ and having poor glycemic control were found to be significantly associated with DM complication.

### Recommendations

Based on the findings of this study; provision of health education, self-care practice and early diagnose and proper management of patient condition should be strengthened.

## Limitation of the study

The study design was cross sectional nature of the study so it is snap shot and could not confirm cause and effect relationship.

## Data Availability

The data supporting the finding had attached to editorial office if necessarily.

## Abbreviations

DM: diabetes mellitus
FBS: fasting blood sugar
RBS: random blood sugar
WHO: World Health Organization
JUSH: Jimma University Specialized Hospital
IDF: International Diabetes Federation
